# Supplementary material for: Phase transition and remodeling complex assembly are important for SS18-SSX oncogenic activity in synovial sarcomas
Source: Nat Commun. 2022 May 18;13:2724. doi: 10.1038/s41467-022-30447-9 (PMC9117659; doi:10.1038/s41467-022-30447-9)
Supplement: Supplementary file 1 — Supplementary Information [file 41467_2022_30447_MOESM1_ESM.pdf]

Supplementary Figure 1

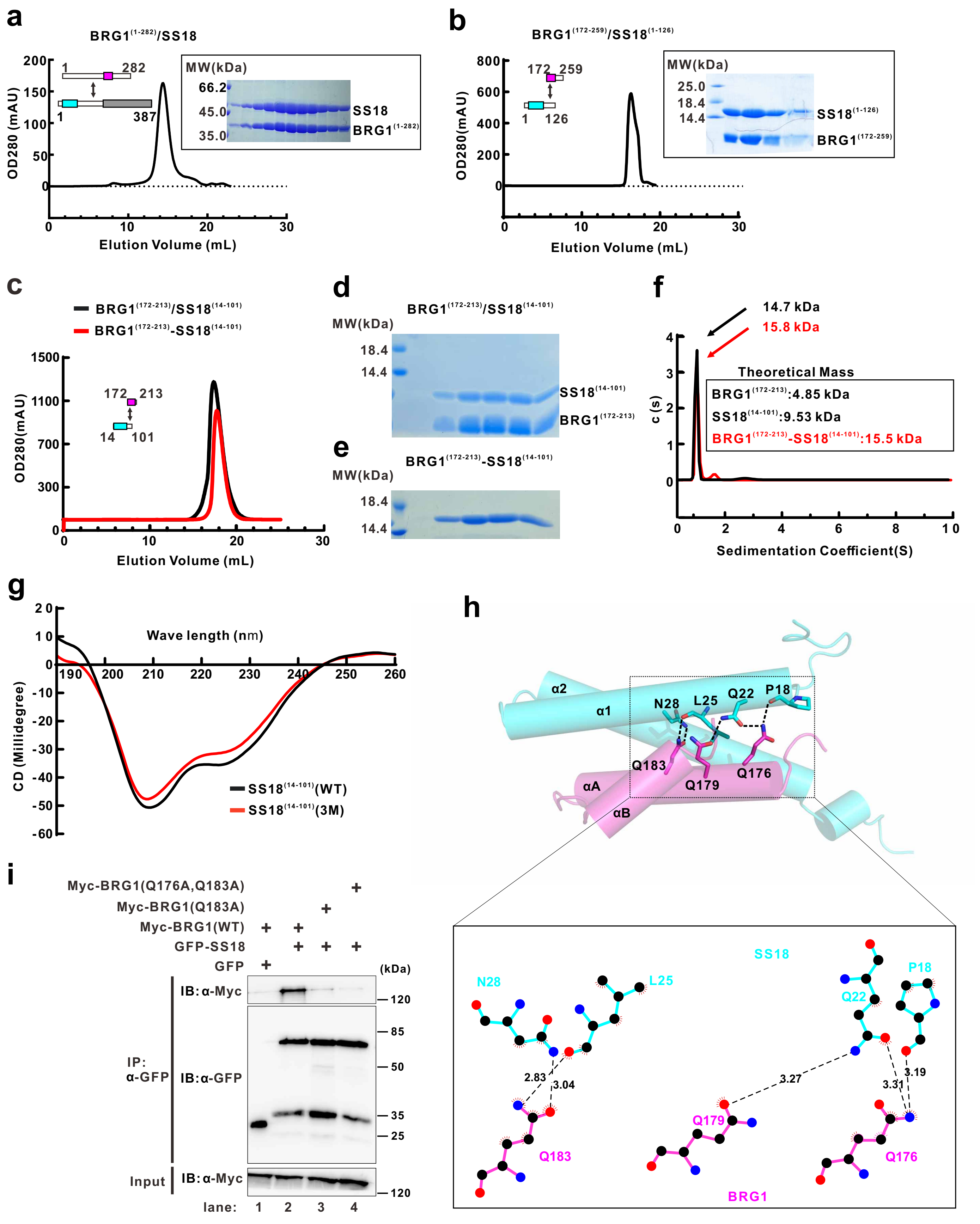

Supplementary Figure 1. Human BRG1/SS18 subcomplex adopts a heterodimer with a 1:1 stoichiometry. Analytical gel filtration profile and SDS-PAGE of the recombinant protein BRG1$^{(1\text{-}282)}$/SS18 **(a)**, or BRG1$^{(172\text{-}259)}$/SS18$^{(1\text{-}126)}$ **(b)**, by co-expression. **(c)** Analytical gel filtration profiles of the BRG1$^{(172\text{-}213)}$/SS18$^{(14\text{-}101)}$ complex and the BRG1$^{(172\text{-}213)}$-SS18$^{(14\text{-}101)}$ protein. "/" denotes protein complex with separate chains, while "-" denotes protein in a single-chain fusion. SDS-PAGEs of recombinant proteins BRG1$^{(172\text{-}213)}$/SS18$^{(14\text{-}101)}$ **(d)** and BRG1$^{(172\text{-}213)}$-SS18$^{(14\text{-}101)}$ **(e)** after gel filtration chromatography shown in (c). **(f)** The molecular weights of the purified proteins were measured by sedimentation velocity (SV) analytical ultracentrifugation. c(s) distributions from the SV runs for the BRG1$^{(172\text{-}213)}$/SS18$^{(14\text{-}101)}$ (1.0 mg/mL, black line) and BRG1$^{(172\text{-}213)}$-SS18$^{(14\text{-}101)}$ (1.0 mg/mL, red line). **(g)** Circular dichroism spectra of SS18$^{(14\text{-}101)}$ (WT) (0.1 mg/mL, black line) or SS18$^{(14\text{-}101)}$ (3M) (0.1 mg/mL, red line) in 20 mM HEPES, pH 7.0 and 50 mM NaCl. **(h)** Ligplot diagram of hydrogen-bonding interactions between SS18 and BRG1. Hydrogen bonds are shown as black dotted lines. The numbers above the lines represent distance and the unit is Å. Black solid dots represent carbon atoms, blue solid dots represent nitrogen atoms, and red solid dots represent oxygen atoms. **(i)** Co-IP experiments testing the interaction between BRG1 wild-type (WT) or mutants and SS18. Extracts were prepared from HEK293T cells transfected with various combinations of plasmids, as indicated. The bottom panel shows 3% of the Myc-BRG1 as input for each IP.

Supplementary Figure 2

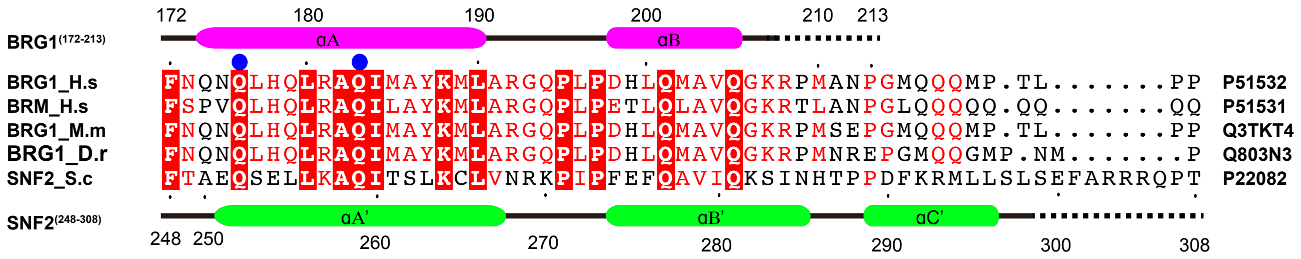

Supplementary Figure 2. Structure-based sequence alignment of BRG1 from different species. In this alignment, the secondary structures of human BRG1$^{(172\text{-}213)}$ and yeast SNF2$^{(248\text{-}308)}$ are shown at the top and bottom, respectively, according to the crystal structures of the BRG1$^{(172\text{-}213)}$-SS18$^{(14\text{-}101)}$ complex and the SNF11$^{(38\text{-}169)}$/SNF2$^{(248\text{-}308)}$ complex, and conserved residues are shaded in red. The highly conserved residues, which are mutated in BRG1(Q176A) or BRG1(Q183A) and SNF2(Q252A) or SNF2(Q259A), are indicated with blue spheres. Species abbreviations: H.s, *Homo sapiens*; M.m, *Mus musculus*; D.r, *Danio rerio*; S.c, *Saccharomyces cerevisiae*. The GenBank numbers are shown at the end of each alignment.

Supplementary Figure 3

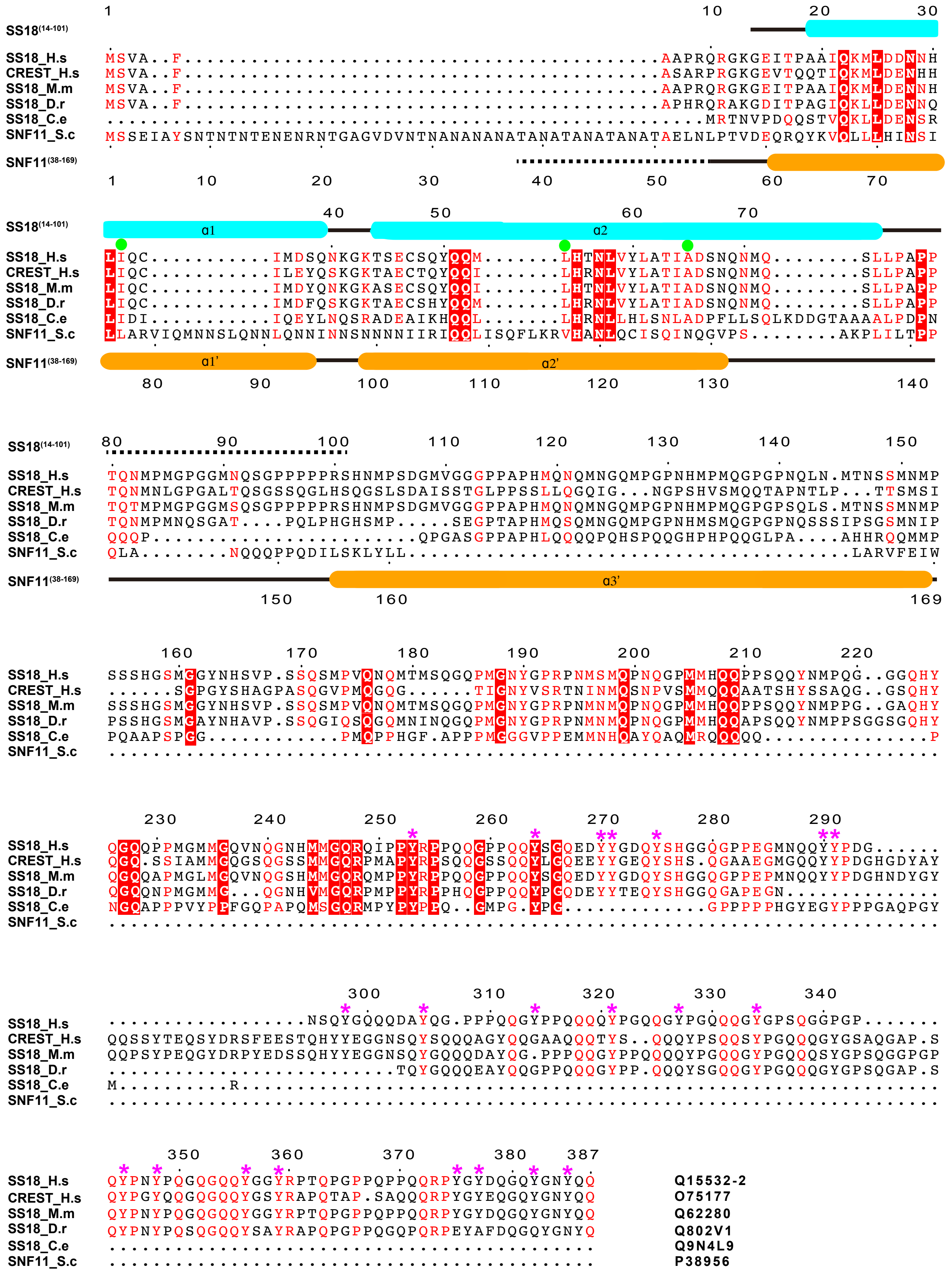

Supplementary Figure 3. Structure-based sequence alignment of SS18 from different species. In this alignment, the secondary structures of human SS18$^{(14\text{-}101)}$ and yeast SNF11$^{(38\text{-}169)}$ are shown at the top and bottom, respectively, and conserved residues are shaded in red. Amino acids substitutions I32E, L54E, and A65E of the mutant SS18(3M) or SS18(3M)-SSX1, are indicated with green spheres. 21 and 19 tyrosine residues, which are mutated in SS18(Y21S) and SS18(Y19S)-SSX1, respectively, are indicated with magenta asterisks. SS18(Y21S) contains the same 19 tyrosine mutations as that of SS18(Y19S)-SSX1 and two carboxyl-terminal mutations Y382 and Y385. Species abbreviations: H.s, *Homo sapiens*; M.m, *Mus musculus*; D.r, *Danio rerio*; C.e, *Caenorhabditis elegans*; S.c, *Saccharomyces cerevisiae*. The GenBank numbers are shown at the end of each alignment.

Supplementary Figure 4

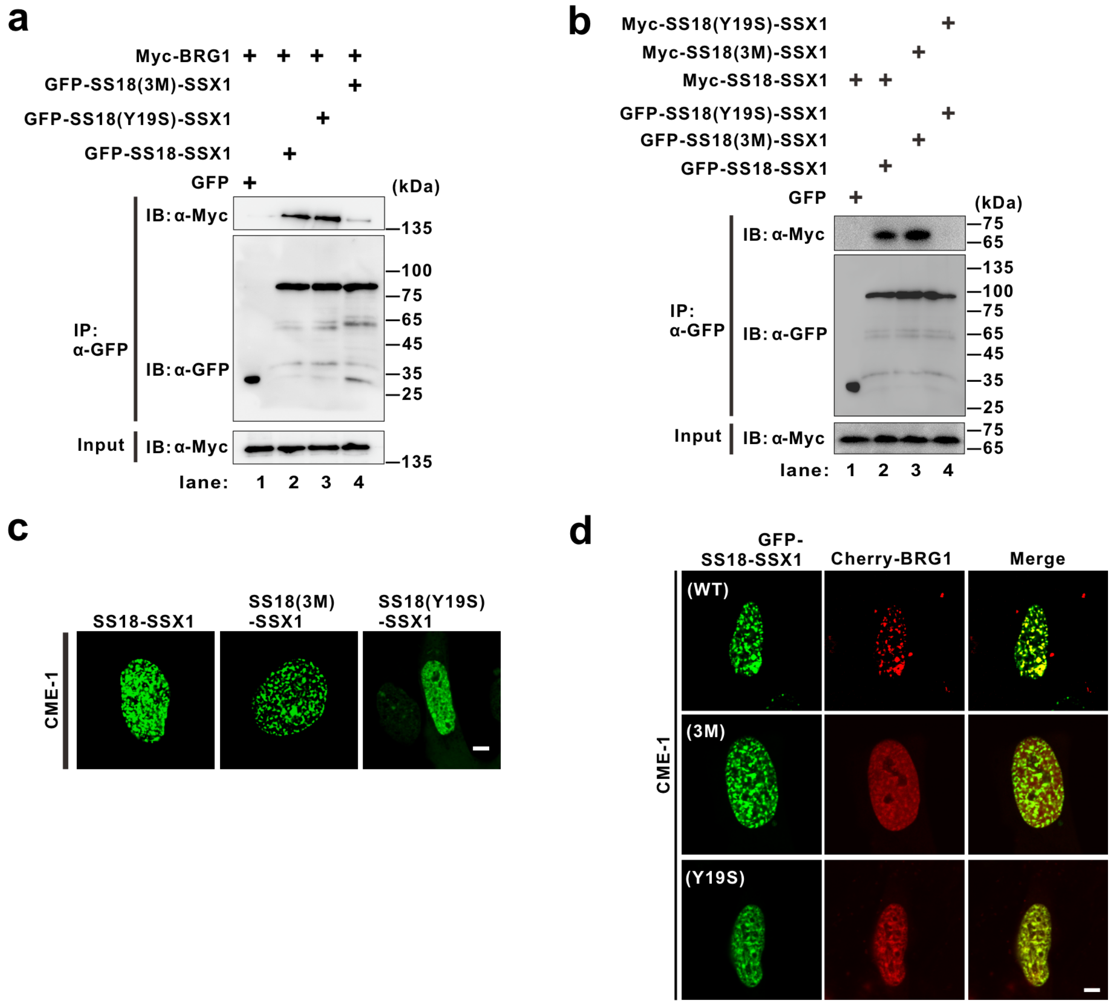

Supplementary Figure 4. SS18-SSX1 binds to BRG1 and is capable of self-association. (a) Co-IP experiments testing the interaction between SS18-SSX1 wild-type (WT) or mutants and BRG1. Extracts were prepared from HEK293T cells transfected with various combinations of plasmids, as indicated. The bottom panel shows 3% of the Myc-BRG1 as input for each IP. **(b)** Co-IP experiments testing the self-association ability of SS18-SSX1 or its mutants. Extracts were prepared from HEK293T cells transfected with various combinations of plasmids, as indicated. The bottom panel shows 3% of the Myc-SS18-SSX1 as input for each IP. **(c)** Live-cell imaging for GFP-SS18-SSX1, GFP-SS18(3M)-SSX1, and GFP-SS18(Y19S)-SSX1 in CME-1 cells. The scale bar indicates 5 μm. **(d)** Co-expression of GFP-SS18-SSX1 or mutants and Cherry-BRG1 in CME-1 cells. The scale bar indicates 5 μm.

Supplementary Figure 5

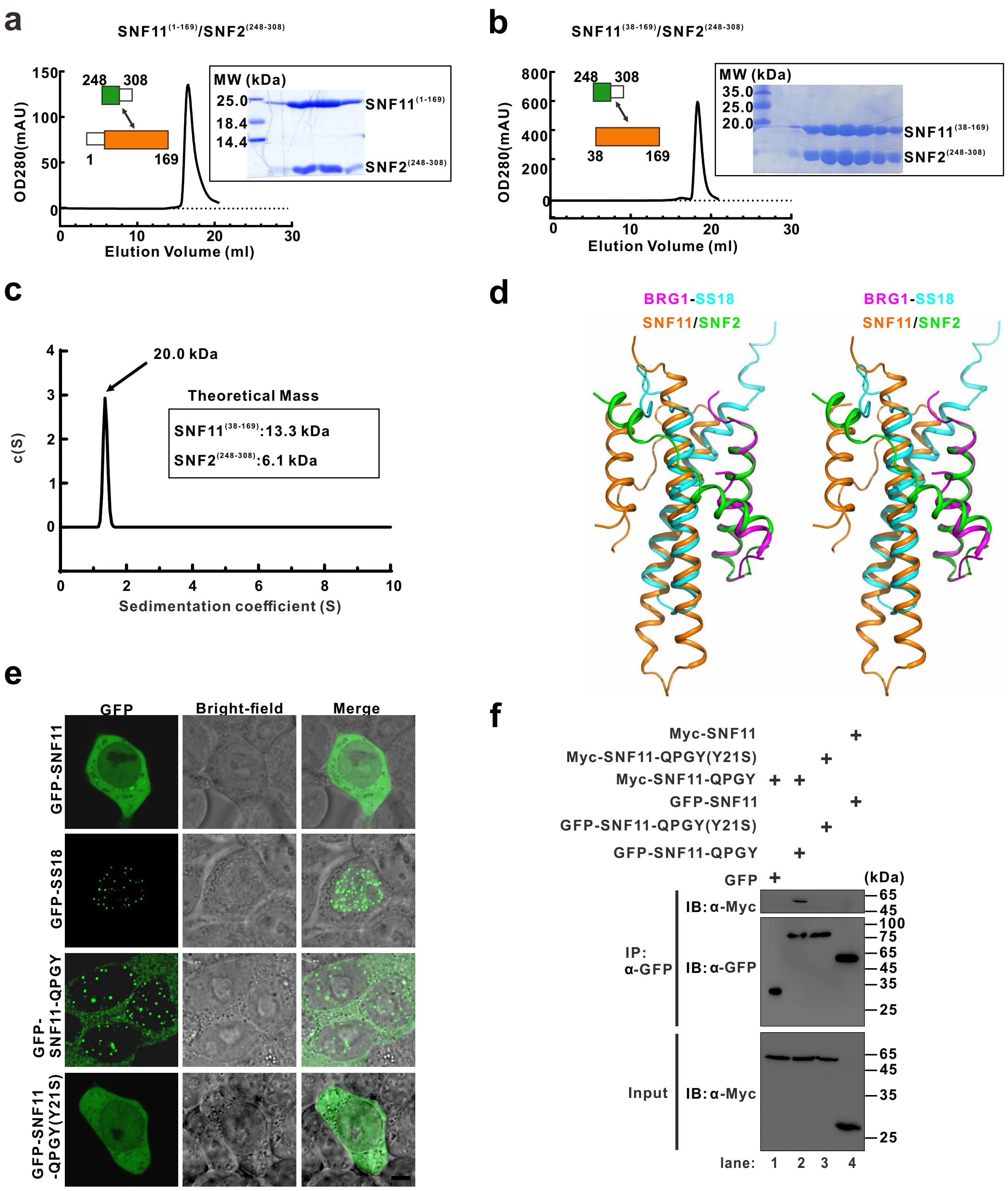

Supplementary Figure 5. Yeast SNF11/SNF2 subcomplex adopts a heterodimer with 1:1 stoichiometry. Analytical gel filtration profile and SDS-PAGE of the complex SNF11$^{(1-169)}$/SNF2$^{(248-308)}$ **(a)**, or SNF11$^{(38-169)}$/SNF2$^{(248-308)}$ **(b)**. **(c)** The molecular weights of the purified proteins were measured by sedimentation velocity (SV) analytical ultracentrifugation. c(s) distributions from the SV run for SNF11$^{(38-169)}$/SNF2$^{(248-308)}$ complex (1.0 mg/ml). **(d)** Stereoview by wall-eye mode showing structure comparison of yeast SNF11$^{(38-169)}$ (orange)/SNF2$^{(248-308)}$ (green) complex and human SS18$^{(14-101)}$ (cyan)-BRG1$^{(172-213)}$ (magenta) complex. **(e)** Live-cell imaging (GFP) and concurrent phase-contrast imaging for GFP-SNF11, GFP-SS18, GFP-SNF11-QPGY, and GFP-SNF11-QPGY(Y21S) in HEK293T cells. The scale bar indicates 5 μm. **(f)** Co-IP experiments testing the self-association ability of SNF11-QPGY, SNF11-QPGY(Y21S) and SNF11. Extracts were prepared from HEK293T cells transfected with various combinations of plasmids, as indicated. The bottom panel shows 3% of the Myc-labeled protein as input for each IP.

Supplementary Figure 6

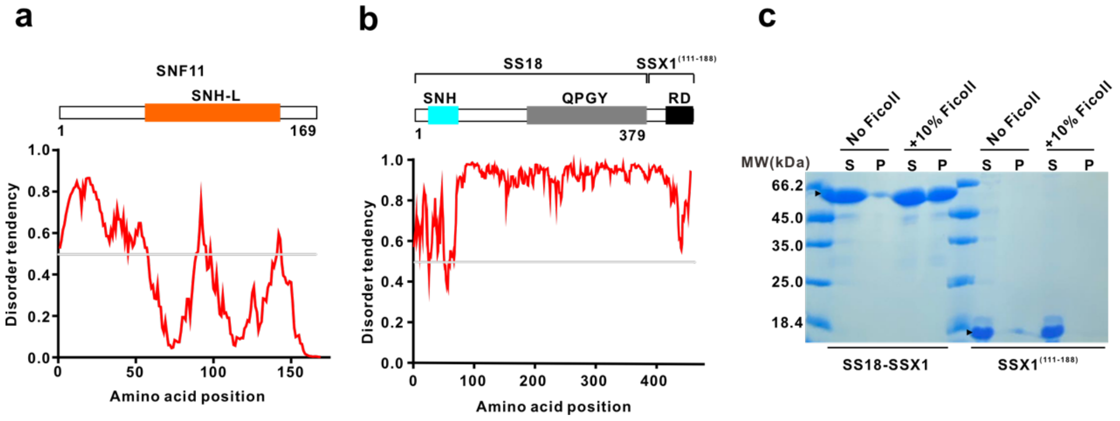

Supplementary Figure 6. SSX1$^{(111\text{-}188)}$ cannot occur phase separation in vitro. (a-b) Domain structure and IUPred assigned scores of intrinsically disordered tendencies of SNF11 **(a)** and SS18-SSX1 **(b)**. A score (shown as red line) of more than 0.5 indicates disordered. **(c)** Representative SDS-PAGE analysis of the sedimentation assay of SS18-SSX1 or SSX1$^{(111\text{-}188)}$ protein in the buffer (50 mM Tris- HCl, pH7.5 and 150 mM NaCl) with or without 10% Ficoll. The concentration of each protein is 60 µM. The black arrowhead denotes the protein band of SS18 -SSX1 or SSX1$^{(111\text{-}188)}$. S, supernatant; P, pellet.

Supplementary Table 1. Data collection and refinement statistics

	BRG1-SS18 Native	BRG1-SS18 Se-Met	SNF11/SNF2 Native	SNF11/SNF2 HgAc2
**Data collection**				
Space group	*F222*	*P41212*	*P212121*	*P212121*
Unit cell (a, b, c, Å):	101.607, 105.829, 225.857	99.856, 99.856, 74.562	47.445, 63.539, 118.466	47.571, 63.417, 119.177
Wavelength (Å)	0.9788	0.9789	0.9789	0.9793
Resolution range (Å)	50.0-2.39(2.43-2.39) [a]	50.0-2.97(3.02-2.97) [a]	50.0-2.15(2.19-2.15) [a]	40.0-2.47(2.51-2.47) [a]
No. of unique reflections	24,275	8,283	20,093	13,596
Redundancy	13.1(11.1) [a]	24.2(17.5) [a]	4.8(4.6) [a]	11.1(7.9) [a]
$\boldsymbol{R}_{\mathrm{sym}}$ (%)[b]	6.3(95.3) [a]	15.2(137.9) [a]	10.4(75.8) [a]	17.8(58.7) [a]
$\boldsymbol{I/\sigma}$	39.2(2.3) [a]	29.0(2.0) [a]	14.8(2.1) [a]	21.9(2.4) [a]
Completeness (%)	99.9(100.0) [a]	100.0(100.0) [a]	99.7(99.9) [a]	99.9(98.5) [a]
**Refinement**				
Resolution range (Å)	28.2-2.39		44.1-2.15	
$\boldsymbol{R}_{\mathrm{crystal}}$ (%)[c]	20.45		19.42	
$\boldsymbol{R}_{\mathrm{free}}$ (%)[d]	25.04		25.99	
$\mathrm{RMSD}_{\mathrm{bond}}$ (Å)	0.008		0.008	
$\mathrm{RMSD}_{\mathrm{angle}}$(°)	0.885		0.932	
Number of				
Protein atoms/Water	3,393/154		2,564/140	
Residues in (%)				
Favored/Allowed/Disallowed	96.7/3.3/0		99.3/0.7/0	
Average B factor of Protein	43.66		29.42	

[a] the highest resolution shell. [b] $R_{sym} = \sum_j |\langle I \rangle - I_j| / \sum \langle I \rangle$. [c] $\boldsymbol{R}_{\mathrm{crystal}} = \sum_{hkl} |F_{obs} - F_{calc}| / \sum_{hkl} F_{obs}$. [d] $\boldsymbol{R}_{\mathrm{free}}$, calculated the same as $\boldsymbol{R}_{\mathrm{crystal}}$, but from a test set containing 5% of data excluded from the refinement calculation. The PDB entry codes of SS18/BRG1 and SNF11/SNF2 complexes are 7VRB and 7VRC, respectively.

Supplementary Table 2. Primers used in this study.

Constructs name	primer sequence 5'-3'
Figs. 1 and 3; Supplementary Fig. 1	
SS18	F:CGCAGATCTATGTCTGTGGCTTTCGCGGCCCCG<br>R:CAGCTCGAGTCACTGCTGGTAATTTCCATACTGT
SS18$^{(1-126)}$	F:CGCAGATCTATGTCTGTGGCTTTCGCGGCCCCG<br>R:CAGCTCGAGTCACTGGCCGTTCATCTGGTTCTGC
SS18$^{(14-101)}$	F:CGCAGATCTGGGGAGATCACTCCCGCTGCGATTC<br>R:CAGCTCGAGTCAAGAGCGTGGAGGTTGTGGGTG
SS18$^{(14-101)}$ (3M)	F:CGCAGATCTGGGGAGATCACTCCCGCTGCGATTC<br>R:CAGCTCGAGTCAAGAGCGTGGAGGTTGTGGGTG
BRG1	F:ATGGAGGCCCGAATTCGGTCGACCATGTCCACTC<br>CAGACCCACCCCTGG<br>R:GCGGCCGCGGTACCTCGAGAGATCTTCAGTCTTC<br>TTCGCTGCCACTTCC
BRG1(Q183A)	F1:ATGGAGGCCCGAATTCGGTCGACCATGTCCACT<br>CCAGACCCACCCCTGG<br>R1:GCGGCCGCGGTACCTCGAGAGATCTTCAGTCTT<br>CTTCGCTGCCACTTCC<br>F2:GCTCAGAGCTGCGATCATGGCCTACAAGATGCT<br>R2:AGGCCATGATCGCAGCTCTGAGCTGGTGCAGCT
BRG1<br>(Q176A,Q183A)	F1:ATGGAGGCCCGAATTCGGTCGACCATGTCCACT<br>CCAGACCCACCCCTGG<br>R1:GCGGCCGCGGTACCTCGAGAGATCTTCAGTCTT<br>CTTCGCTGCCACTTCC<br>F2:TAACCAGAACGCGCTGCACCAGCTCAGAGCT<br>R2:GCTGGTGCAGCGCGTTCTGGTTAAATGGGGTTG
BRG1$^{(1-282)}$	F:ACCGGATCCATGTCCACTCCAGACCCACCCCTGG<br>R:ATCAAGCTTTACTTGGGAGGCCCTCCAGGAGG
BRG1$^{(172-259)}$	F:TGAGAATTCTTTAACCAGAACCAGCTGCACCAGC<br>R:ATCAAGCTTTTAGTTGGGCCCTCCCATACCATGA<br>GGC
BRG1$^{(172-213)}$	F:TGAGAATTCTTTAACCAGAACCAGCTGCACCAGC<br>R:ATGGTCGACTTACATCCCGGGCATCGGCCGCTTG
Fig. 2; Supplementary Fig. 5	
SNF11$^{(1-169)}$	F:AACGGATCCATGAGCAGTGAAATTGCCTACTCG<br>R:ATCAAGCTTCTACCATATCTCGAACACTCTTG
SNF11$^{(38-169)}$	F:TGAGAATTCACTGCAAATGCAACTGCAAATGC<br>R:ATCAAGCTTCTACCATATCTCGAACACTCTTG
SNF11-QPGY	F1:AACGGATCCATGAGCAGTGAAATTGCCTACTCG<br>R1:CAGCTCGAGTCACTGCTGGTAATTTCCATACTG<br>T<br>F2:TTCGAGATATGGGGTGGGGGTCCTCCTGCACC<br>R2:GACCCCCACCCCA`TATCTCGAACACTCTTGCC

SNF11-QPGY(Y21S)	F1:AACGGATCCATGAGCAGTGAAATTGCCTACTCG<br>R1:CAGCTCGAGTTACTGCTGGCTATTACCGCTCTGACC<br>F2:TTCGAGATATGGGGTGGGGGTCCTCCTGCACC<br>R2:GACCCCCACCCCATATCTCGAACACTCTTGCC
SNF2	F:ATGGTCGACCATGAACATACCACAGCGTCAATTTAG<br>R:ATGGCGGCCGCCTATACACTCGCTTCTGTCATGC
SNF2(Q259A)	F1:ATGGTCGACCATGAACATACCACAGCGTCAATTTAG<br>R1:ATGGCGGCCGCCTATACACTCGCTTCTGTCATGC<br>F2:GTTAAAGGCCGCAATCACATCTCTAAAATGTCT<br>R2:GAGATGTGATTGCGGCCTTTAACAGTTCGGAT
SNF2 (Q252A,Q259A)	F1:ATGGTCGACCATGAACATACCACAGCGTCAATTTAG<br>R1:ATGGCGGCCGCCTATACACTCGCTTCTGTCATGC<br>F2:TACTGCCGAGGCATCCGAACTGTTAAAGGCCCA<br>R2:ACAGTTCGGATGCCTCGGCAGTAAACATTGTCA
SNF2$^{(248-308)}$	F:CGCAGATCTTTTACTGCCGAGCAATCCGAACTG<br>R:CAGCTCGAGTTAATCTGTGGGTTGTCTTCTTCTCGC
Fig. 4	
SS18(Y21S)	F:CGCAGATCTATGTCTGTGGCTTTCGCGGCCCCG<br>R:CAGCTCGAGTTACTGCTGGCTATTACCGCTCTGACC
SS18(3M)	F1:CGCAGATCTATGTCTGTGGCTTTCGCGGCCCCG<br>R1:CAGCTCGAGTCACTGCTGGTAATTTCCATACTGT<br>F2:CAGTATCAGCAGATGGAACACACAAACTTGGTATACCTTGCTACAATAGAAGATTCTAATCAAAATATGCAGTC<br>R2:GATTAGAATCTTCTATTGTAGCAAGGTATACCAAGTTTGTGTGTTCCATCTGCTGATACTGAGAACACTCTGAGG<br>F3:TGACAATAACCATCTTGAACAGTGTATAATGGACTCTCAGAAT<br>R3:GTCCATTATACACTGTTCAAGATGGTTATTGTCATCCAACA
Fig. 5; Supplementary Fig. 4	
SS18-SSX1	F1:CGCAGATCTATGTCTGTGGCTTTCGCGGCCCCG<br>R1:ATCCTCGAGTTATTCATCATCTTCTTCCGGATCG<br>F2:GACCAGATTATGCCGAAAAAACCGGCC

	R2:CATAATCTGGTCATATCCATAAGGC
SS18(Y19S)-SSX1	F1:CGCAGATCTATGTCTGTGGCTTTCGCGGCCCCG<br>R1:ATCCTCGAGTTATTCATCATCTTCTTCCGGATCG<br>F2:GATCAAATTATGCCGAAAAAACCGGCCGAGG<br>R2:CATAATTTGATCGCTGCCACTCGGACGCTGC
SS18(3M)-SSX1	F1:CGCAGATCTATGTCTGTGGCTTTCGCGGCCCCG<br>R1:ATCCTCGAGTTATTCATCATCTTCTTCCGGATCG<br>F2:GATCAAATTATGCCGAAAAAACCGGCCGAGG<br>R2:CATAATCTGGTCATATCCATAAGGC
Supplementary Fig. 6	
SSX1$^{(111-188)}$	F:TGAGAATTCATTATGCCGAAAAAACCGGCC<br>R:ATCAAGCTTTTATTCATCATCTTCTTCCGGATCG
Fig. 6	
Myc-SS18-SSX1	F:GCCACTAGTATGGCATCAATGCAGAAGCTGATCTCAGAGGAGGACCTGCTTATGGCCATGTCTGTGGCTTTCGC<br>R:CTCGAATTCTTATTCATCATCTTCTTCCGGATCGCTG
Myc-SS18(3M)-SSX1	F:GCCACTAGTATGGCATCAATGCAGAAGCTGATCTCAGAGGAGGACCTGCTTATGGCCATGTCTGTGGCTTTCGC<br>R:CTCGAATTCTTATTCATCATCTTCTTCCGGATCGCTG
Myc-SS18(Y19S)-SSX1	F:GCCACTAGTATGGCATCAATGCAGAAGCTGATCTCAGAGGAGGACCTGCTTATGGCCATGTCTGTGGCTTTCGC<br>R:CTCGAATTCTTATTCATCATCTTCTTCCGGATCGCTG
