# Supplementary material for: Phase transition and remodeling complex assembly are important for SS18-SSX oncogenic activity in synovial sarcomas
Source: Nat Commun. 2022 May 18;13:2724. doi: 10.1038/s41467-022-30447-9 (PMC9117659; doi:10.1038/s41467-022-30447-9)
Supplement: Supplementary file 3 — Reporting Summary [file 41467_2022_30447_MOESM3_ESM.pdf]

nature portfolio

Corresponding author(s): Hao Zhou, Jiafu Long

Last updated by author(s): Apr 19, 2022

Reporting Summary

Nature Portfolio wishes to improve the reproducibility of the work that we publish. This form provides structure for consistency and transparency in reporting. For further information on Nature Portfolio policies, see our Editorial Policies and the Editorial Policy Checklist.

Statistics

For all statistical analyses, confirm that the following items are present in the figure legend, table legend, main text, or Methods section.

n/a	Confirmed	
☐	☒	The exact sample size (*n*) for each experimental group/condition, given as a discrete number and unit of measurement
☐	☒	A statement on whether measurements were taken from distinct samples or whether the same sample was measured repeatedly
☐	☒	The statistical test(s) used AND whether they are one- or two-sided<br>*Only common tests should be described solely by name; describe more complex techniques in the Methods section.*
☒	☐	A description of all covariates tested
☒	☐	A description of any assumptions or corrections, such as tests of normality and adjustment for multiple comparisons
☐	☒	A full description of the statistical parameters including central tendency (e.g. means) or other basic estimates (e.g. regression coefficient) AND variation (e.g. standard deviation) or associated estimates of uncertainty (e.g. confidence intervals)
☐	☒	For null hypothesis testing, the test statistic (e.g. F, t, r) with confidence intervals, effect sizes, degrees of freedom and P value noted<br>*Give P values as exact values whenever suitable.*
☒	☐	For Bayesian analysis, information on the choice of priors and Markov chain Monte Carlo settings
☒	☐	For hierarchical and complex designs, identification of the appropriate level for tests and full reporting of outcomes
☒	☐	Estimates of effect sizes (e.g. Cohen's d, Pearson's r), indicating how they were calculated

Our web collection on statistics for biologists contains articles on many of the points above.

Software and code

Policy information about availability of computer code

Data collection	1. All diffraction data were collected at the Shanghai Synchrotron Radiation Facility (SSRF) on beamlines BL19U1.<br>2. Confocal images were taken by Zeiss LSM 710 microscope and then the data were collected by the Zen black v2011 software.<br>3. Observation and characterization of droplets were carried out on a fluorescence microscope (LEICA CTR5000), and then the data were collected by the Leica Application Suite v4.4.0 software.<br>4. EdU assay, cell migration, and invasion assay were observed with a fluorescence microscope system Nikon ECLIPSE Ti-S, and then the data were collected by the NIS-Elements F v4.0 software.
Data analysis	1. The diffraction data were processed using the HKL2000 v714 software package. Phasing and initial model building of human complex crystal structure was determined by single wavelength anomalous dispersion (SAD) using PHENIX v1.15-2155 AutoSol wizard and AutoBuild wizard, respectively. The initial phases and models of yeast complex were determined by SAD using the Shelx C/D/E program in CCP4i v7.0.073. Then, the initial models were further rebuilt and adjusted manually with the Coot v0.8.3 program and were refined by refinement program in Phenix v1.15-2155. The final model was further validated using MolProbity in Phenix v1.15-2155. All structural figures were prepared using PyMOL v1.6 and the planar graph of protein-protein interaction were generated by LigPlot+ v2.2 software.<br>2. The SV data were analyzed using the SEDFIT program v14.0 .<br>3. The data of sedimentation experiment analysis, EdU assay, cell migration, and invasion assay were analyzed by Image J v1.8.0, GraphPad Prism v8.0.

For manuscripts utilizing custom algorithms or software that are central to the research but not yet described in published literature, software must be made available to editors and reviewers. We strongly encourage code deposition in a community repository (e.g. GitHub). See the Nature Portfolio guidelines for submitting code & software for further information.

Data

Policy information about availability of data

All manuscripts must include a data availability statement. This statement should provide the following information, where applicable:

- Accession codes, unique identifiers, or web links for publicly available datasets
- A description of any restrictions on data availability
- For clinical datasets or third party data, please ensure that the statement adheres to our policy

The nucleotide sequence of yeast SNF11 and SNF2 were obtained from Saccharomyces cerevisiae genome databases with SGD ID of S00002480 and S00005816, respectively. The atomic coordinates and structure factors data for the crystal structure of the BRG1/SS18 and SNF11/SNF2 complex have been deposited in the Protein Data Bank database under accession code 7VRB [http://doi.org/10.2210/pdb7VRB/pdb] and 7VRC [http://doi.org/10.2210/pdb7VRC/pdb], respectively. The source data underlying Figs. 1f, 2d, 3a, 3d, 3e, 3h, 4a-c, 5c, 5d, 5g, 6a, 6c, 6f, and 6h and Supplementary Figs. 1a, 1b, 1d, 1e, 1i, 4a, 4b, 5a, 5b, 5f, 6c are provided as a Source Data file. Source data are provided with this paper. The authors declare that all data supporting the finding of this study are available within this article and its supplementary information files.

Field-specific reporting

Please select the one below that is the best fit for your research. If you are not sure, read the appropriate sections before making your selection.

[x] Life sciences [ ] Behavioural & social sciences [ ] Ecological, evolutionary & environmental sciences

For a reference copy of the document with all sections, see nature.com/documents/nr-reporting-summary-flat.pdf

Life sciences study design

All studies must disclose on these points even when the disclosure is negative.

Sample size	None
Data exclusions	None
Replication	Each experiment of analytical gel filtration and SDS-PAGE, Co-IP assays and western blot assays were performed twice independently with similar results. For the data of phase separation, droplet formation assays in vitro and confocal images of living cells were acquired from three independent experiment, and more than 6 images were taken for each sample. They showed similar results. Sedimentation experiments were repeated three times independently with similar results. EdU assays were performed with 5 independent experiments, Cell migration and invasion assays were performed with 3 independent experiments. All attempts at replication were successful.
Randomization	None
Blinding	The investigations were blinded to group allocation during data collection and analysis.

Reporting for specific materials, systems and methods

We require information from authors about some types of materials, experimental systems and methods used in many studies. Here, indicate whether each material, system or method listed is relevant to your study. If you are not sure if a list item applies to your research, read the appropriate section before selecting a response.

Materials & experimental systems

n/a	Involved in the study	
[ ]	[x]	Antibodies
[ ]	[x]	Eukaryotic cell lines
[x]	[ ]	Palaeontology and archaeology
[x]	[ ]	Animals and other organisms
[x]	[ ]	Human research participants
[x]	[ ]	Clinical data
[x]	[ ]	Dual use research of concern

Methods

n/a	Involved in the study	
[x]	[ ]	ChIP-seq
[x]	[ ]	Flow cytometry
[x]	[ ]	MRI-based neuroimaging

Antibodies

Antibodies used	Anti-Myc, Sigma-Aldrich, M4439-100UL, clone 9E10; Anti-GFP, Sigma-Aldrich, G1544-100UG; Goat anti-mouse IgG-HRP, Santa cruz, sc-2005; Goat anti-rabbit IgG-HRP, Santa cruz, sc-2004; HRP-conjugated GAPDH, Proteintech, HRP-60004, clone 1E6D9.

Validation	Mouse Anti-Myc, Sigma-Aldrich, M4439-100UL, clone 9E10, western blotting (WB) validation. http://www.sigmaaldrich.cn/CN/zh/product/sigma/m4439?context=product<br>Mouse Anti-GFP, Sigma-Aldrich, G1544-100UG, western blotting (WB) validation. http://www.sigmaaldrich.cn/CN/zh/product/sigma/m1544?context=product<br>Goat anti-mouse IgG-HRP, Santa cruz, sc-2005, western blotting (WB) validation. https://www.scbt.com/p/goat-anti-mouse-igg-hrp?requestFrom=search<br>Goat anti-rabbit IgG-HRP, Santa cruz, sc-2004, western blotting (WB) validation. https://www.scbt.com/p/goat-anti-mouse-igg-hrp?requestFrom=search<br>HRP-conjugated GAPDH, Proteintech, HRP-60004, clone 1E6D9, western blotting (WB) validation. http://www.ptglab.com/products/GAPDH-Antibody-HRP-60004.htm

Eukaryotic cell lines

Policy information about cell lines

Cell line source(s)	Human embryonic kidney (HEK) 293T cell line (CBTCCCAS, GNHu17), Henrietta Lacks (HeLa) cell line (CBTCCCAS, TCHu187), and mouse embryonic fibroblast cell line NIH3T3 (CBTCCCAS, SCSP-515) were obtained from Cell bank of Type Culture Collection of Chinese Academy of Science (CBTCCCAS). Human synovial sarcoma cell line HS-SY-II was originally from Hiroshi Sonobe, Department of Pathology, Kochi Medical School, Nankoku, Japan. Human synovial sarcoma cell line CME-1 was originally from Nicolo Riggi, Division of Experimental Pathology, Institute of Pathology, Centre Hospitalier Universitaire Vaudois and University of Lausanne, Lausanne, Switzerland.
Authentication	All the cell lines used were validated by suppliers.
Mycoplasma contamination	All of the cell lines have been confirmed as mycoplasma contamination free.
Commonly misidentified lines<br>(See ICLAC register)	None found in the ICLAC database.
